# Enhancers associated with unstable RNAs are rare in plants

**DOI:** 10.1038/s41477-024-01741-9

**Published:** 2024-07-30

**Authors:** Bayley R. McDonald, Colette L. Picard, Ian M. Brabb, Marina I. Savenkova, Robert J. Schmitz, Steven E. Jacobsen, Sascha H. Duttke

**Affiliations:** 1 School of Molecular Biosciences, College of Veterinary Medicine, Washington State University, Pullman, WA USA; 2 Department of Molecular Cell and Developmental Biology, University of California at Los Angeles, Los Angeles, CA USA; 3 Department of Genetics, University of Georgia, Athens, GA USA; 4 Howard Hughes Medical Institute, University of California at Los Angeles, Los Angeles, CA USA

**Keywords:** Plant molecular biology, Transcriptomics

## Abstract

Unstable transcripts have emerged as markers of active enhancers in vertebrates and shown to be involved in many cellular processes and medical disorders. However, their prevalence and role in plants is largely unexplored. Here, we comprehensively captured all actively initiating (nascent) transcripts across diverse crops and other plants using capped small (cs)RNA sequencing. We discovered that unstable transcripts are rare in plants, unlike in vertebrates, and when present, often originate from promoters. In addition, many ‘distal’ elements in plants initiate tissue-specific stable transcripts and are likely bona fide promoters of as-yet-unannotated genes or non-coding RNAs, cautioning against using reference genome annotations to infer putative enhancer sites. To investigate enhancer function, we integrated data from self-transcribing active regulatory region (STARR) sequencing. We found that annotated promoters and other regions that initiate stable transcripts, but not those marked by unstable or bidirectional unstable transcripts, showed stronger enhancer activity in this assay. Our findings underscore the blurred line between promoters and enhancers and suggest that *cis*-regulatory elements can encompass diverse structures and mechanisms in eukaryotes, including humans.

## Abstract (web summary)

Unstable transcripts like enhancer RNAs are common in vertebrates. McDonald et al. show that such unstable transcripts are rare in plants and that promoters can function as potent enhancers, suggesting diverse *cis*-regulatory mechanisms in eukaryotes.

## Main

The discovery of rapidly degraded and often unprocessed RNAs, such as enhancer-associated RNAs in mammals^1,2^, has sparked the ongoing endeavour to demystify their role and potential functions. Methods that capture actively transcribed or ‘nascent’ RNA rather than steady-state transcript levels that are a result of many processes, including initiation, elongation, maturation and decay^3,4^, were instrumental to this research. These approaches have revealed that unstable RNAs are highly prevalent in vertebrates and are involved in many cellular processes and medical disorders^5^. Unstable transcripts have also been shown to impact gene expression by interacting with transcription factors, co-factors or chromatin^6–11^, and influence the three-dimensional structure of the genome^12^.

Distal, bidirectional, unstable transcripts, often referred to as enhancer RNAs (eRNAs), have emerged as preferred markers of active regulatory regions in vertebrates^1,13–15^. These eRNAs are commonly short, non-polyadenylated, unstable and generated from bidirectionally transcribed loci^14^, although some eRNAs are spliced or polyadenylated^14,16,17^. Similarly to vertebrates, plants leverage distal *cis*-regulatory regions, including traditional enhancers^18–21^. Studies in *Arabidopsis thaliana* and wheat have also reported hundreds to tens of thousands of potential loci marked by uni- or bidirectional unstable transcripts^22–26^ but the prevalence and potential roles of unstable transcripts is largely unexplored^23,25,27,28^.

Given the importance of plants as the world’s primary food source and their central role in enlivening and sustaining the environment, it is critical to address this gap in our knowledge. However, high-quality nascent RNA sequencing datasets from plants, and especially nascent transcription start site (TSS) data, are currently rare. Although some groups, including ours, have shown that methods capturing active transcription, including global run-on sequencing (GRO-seq)^23,29,30^, precision run-on sequencing (PRO-seq)^31^ and plant native elongating transcript sequencing (pNET-seq)^30,32^, are feasible in plants, their application is challenging. Plant cell walls, abundant plastids, and secondary metabolites hinder the necessary isolation of pure nuclei and complicate immunoprecipitation steps. In addition, plants have five or more eukaryotic RNA polymerases and multiple phage-like and plastid-encoded prokaryotic RNA polymerases^33^, and traditional run-on sequencing methods capture nascent transcripts from all these RNA polymerases non-specifically, complicating data interpretation^29,34^. Thus, nascent RNA-seq methods have drastically advanced our understanding of unstable transcripts in animals and yeast^4,35–38^, but less so in plants.

**Fig. 1:**
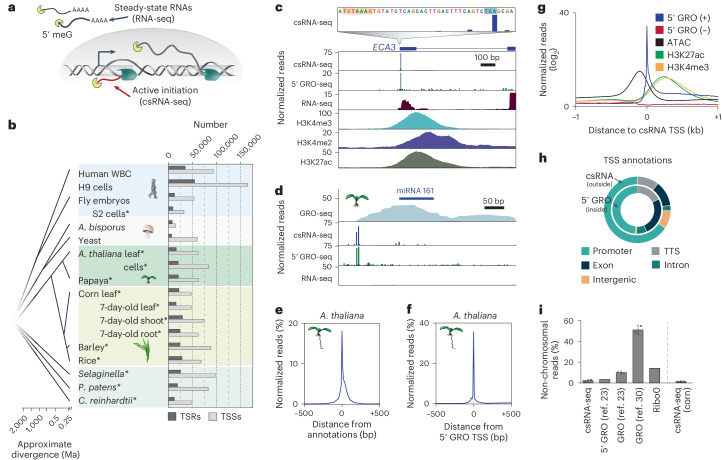
A comprehensive atlas of nascent plant transcription initiation. **a**, Schematic of steady-state RNA, as captured by RNA-seq, and actively initiating or nascent transcripts, captured by csRNA-seq. **b**, Overview of samples studied with the numbers of captured transcription start regions (TSRs), which include promoters and enhancers, and of TSSs. Samples generated in this study are marked with an asterisk (*). **c**, *A. thaliana* *ECA3* loci with csRNA-seq at single-nucleotide resolution and zoomed out, 5′ GRO-seq and histone ChIP-seq data. **d**, *A. thaliana* miRNA 161 cluster. **e**, Normalized distribution of *A. thaliana* csRNA-seq data from leaves relative to TAIR10 TSS annotations. All reads under the graph amount to 100%. **f**, Normalized distribution of csRNA-seq TSSs from *A. thaliana* leaves relative to 5′ GRO-seq TSSs mapped in 6-day-old seedlings. **g**, Distribution of 5′ GRO-seq reads, open chromatin (ATAC-seq) and histone H3 lysine 4 trimethylation (H3K4me3) and H3 lysine 27 acetylation (H3K27ac) relative to csRNA-seq TSSs in *A. thaliana*. **h**, Comparison of annotations of TSSs mapped by 5′ GRO-seq and csRNA-seq in *A. thaliana*. **i**, Percentage of non-chromosomal RNA reads captured by csRNA-seq (0.031% and 0,014%; *n* = 2), GRO-seq (0.109% and 0.09%; *n* = 2)^23^, GRO-seq (0.54% and 0.48%; *n* = 2)^30^, 5′ GRO-seq (0.034%; *n* = 1)^23^, or total RNA-seq (Ribo0, 0.147%; *n* = 1) in *A. thaliana* and maize (csRNA-seq only, 0.009% and 0.01%; *n* = 2). These RNAs are not synthesized by RNA polymerase II or other eukaryotic RNA polymerases. Graphs present the mean with s.d. Ma, million years ago; TTS, transcription termination site; WBC, white blood cells; 5′ meG, 5′ methylguanine.

**Extended Data Fig. 1:**
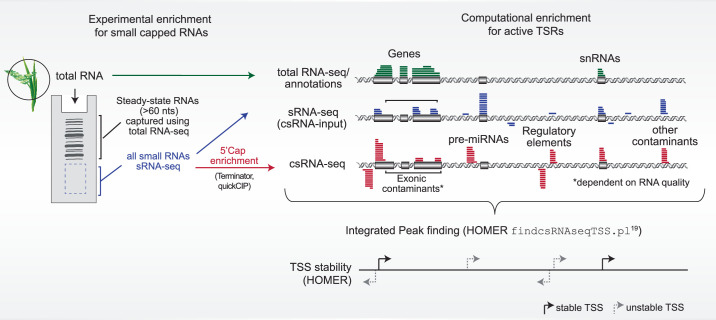
Overview of capped small (cs)RNA-seq. Schematic of experimental and in silico steps performed to enrich actively initiated RNA polymerase II transcripts, which are marked by a 5’cap, from total RNA.

Some of the technical limitations described above can be alleviated by exploiting the RNA-polymerase-II-specific 5′ cap to enrich for nascent RNA polymerase II transcripts and their TSSs^11,36^. Selective sequencing of capped 5′ ends also increases the sensitivity of these methods to detect short, rare and unstable transcripts^39,40^, such as eRNAs^1,2,11^ and promoter-divergent unstable transcripts^41^. We recently developed capped small RNA-seq (csRNA-seq; Extended Data Fig. 1), which leverages these advances to enrich for initiating RNA polymerase II transcripts and capture their TSSs without the need for nuclei isolation, run-on or immunoprecipitation (Fig. 1a)^39^. csRNA-seq is a simple, scalable and cost-efficient protocol that uses 1–3 µg of total RNA, rather than purified nuclei, as input and is compatible with any fresh, frozen, fixed or pathogenic species or tissue^42–45^. Recently, csRNA-seq was shown to effectively detect eRNAs in human cells^40,43,45^.

Here we used csRNA-seq to decipher the prevalence, location and traits of stable and unstable transcripts across different plant tissues, cells and species. Our data suggest that vertebrate-like eRNAs are rare in plants. Instead, promoters were the major source of unstable transcripts. Intriguingly, promoters and open chromatin regions, rather than sites initiating unstable transcription, also showed the strongest enhancer activity in the self-transcribing active regulatory region sequencing (STARR-seq) assay, suggesting that the relationship between unstable transcription and enhancer activity observed in mammals is not conserved in plants.

## Results

### A comprehensive atlas of nascent transcripts in plants

To comprehensively capture active transcription in plants, we performed csRNA-seq on 13 samples from 8 plant species chosen for their agricultural and scientific importance (Fig. 1b and Supplementary Table 1). For comparison, we also performed csRNA-seq on S2 cells from fruit fly (*Drosophila melanogaster*) and integrated published data from fruit fly embryos^46^, rice (*Oryza sativa*, adult leaves)^39^, human white blood cells^45^, human H9 cells^39^ and two types of fungi (common mushroom, *Agaricus bisporus*; yeast, *Saccharomyces cerevisiae*)^47^ (Fig. 1b).

**Extended Data Fig. 2:**
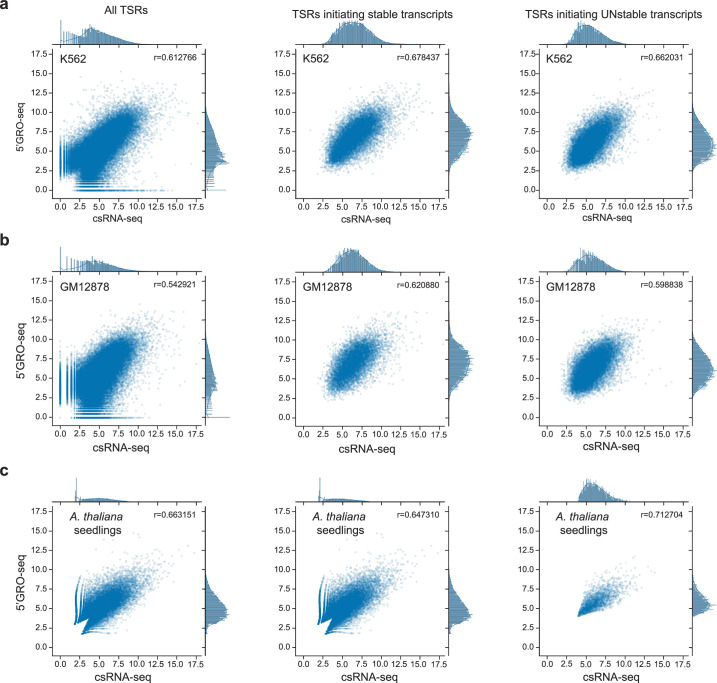
csRNA-seq captures transcription initiation independent of RNA stability. Scatterplots comparing similarity between 5’GRO-seq and csRNA-seq rlog tag normalization for all TSRs, TSRs resulting in stable transcripts, and TSRs resulting in UNstable transcripts for **a**, *Homo sapiens* K562 cells, **b**, *Homo sapiens* GM12878 cells, and **c**, *Arabidopsis thaliana* 6-day-old seedlings.

**Extended Data Fig. 3:**
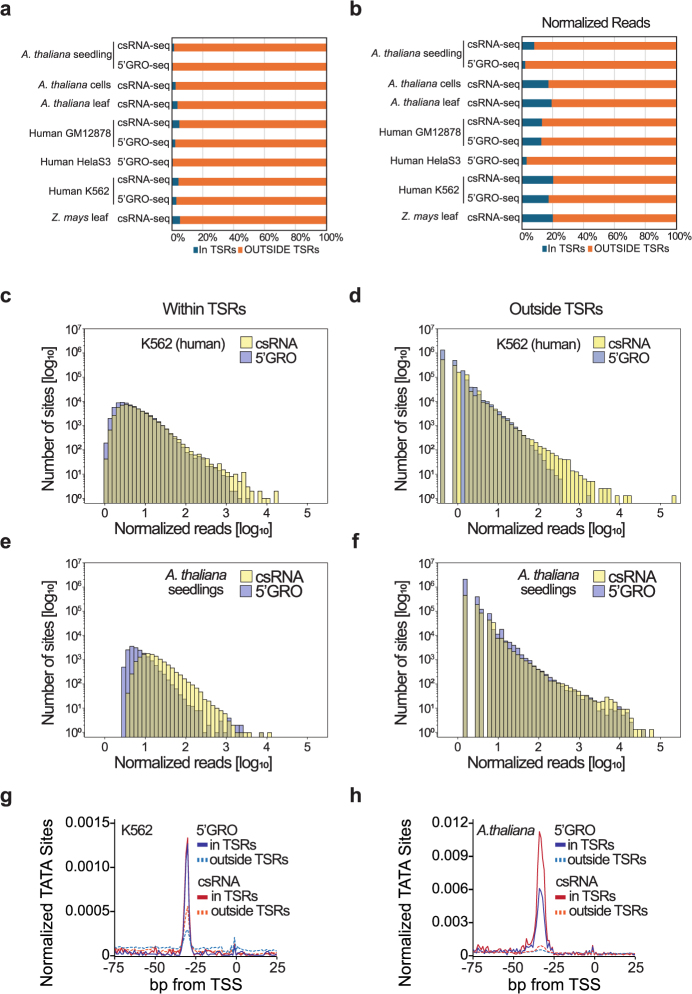
Fine-scale comparison of 5’ ends captured by csRNA-seq and 5’GRO-seq. **a**, Comparison of the percentage of unique positions captured that fell inside or outside of a TSR following peak calling for each library. On average, a higher percentage of tags fell within TSRs for csRNA-seq compared to 5’GRO. **b**, Comparison of the percentage of normalized total read counts captured that fell inside or outside of a TSR following peak calling for each library. **c**-**f**, Comparison of the number of unique sites (y-axis) versus intensity (normalized reads, x-axis) for csRNA-seq and 5’GRO positions from human K562 cells (**c**,**d**) and *A. thaliana* 6-day-old seedlings (**e**,**f**). **c**,**e**, Sites that mapped within TSRs, **d,f**, Sites that mapped outside TSRs. Overall, for these data 5’GRO exhibited enrichment for low signal noise, whereas csRNA-seq showed high signal contaminations, often resulting from small nuclear and small nucleolar RNAs. These abundant steady-state small RNAs are not considered csRNA-seq TSRs due to lack of enrichment over the small RNA-seq utilized as csRNA-seq input. **g**,**h** Frequency analysis of the TATA box motif relative to each unique sequence tag (‘0’) as a biological proxy to measure of noise, as this core promoter element is constrained to the −28 region relative to the TSSs. Data for human K562 cells (**g**) and *A. thaliana* 6-day-old seedlings (**h**).

**Extended Data Fig. 4:**
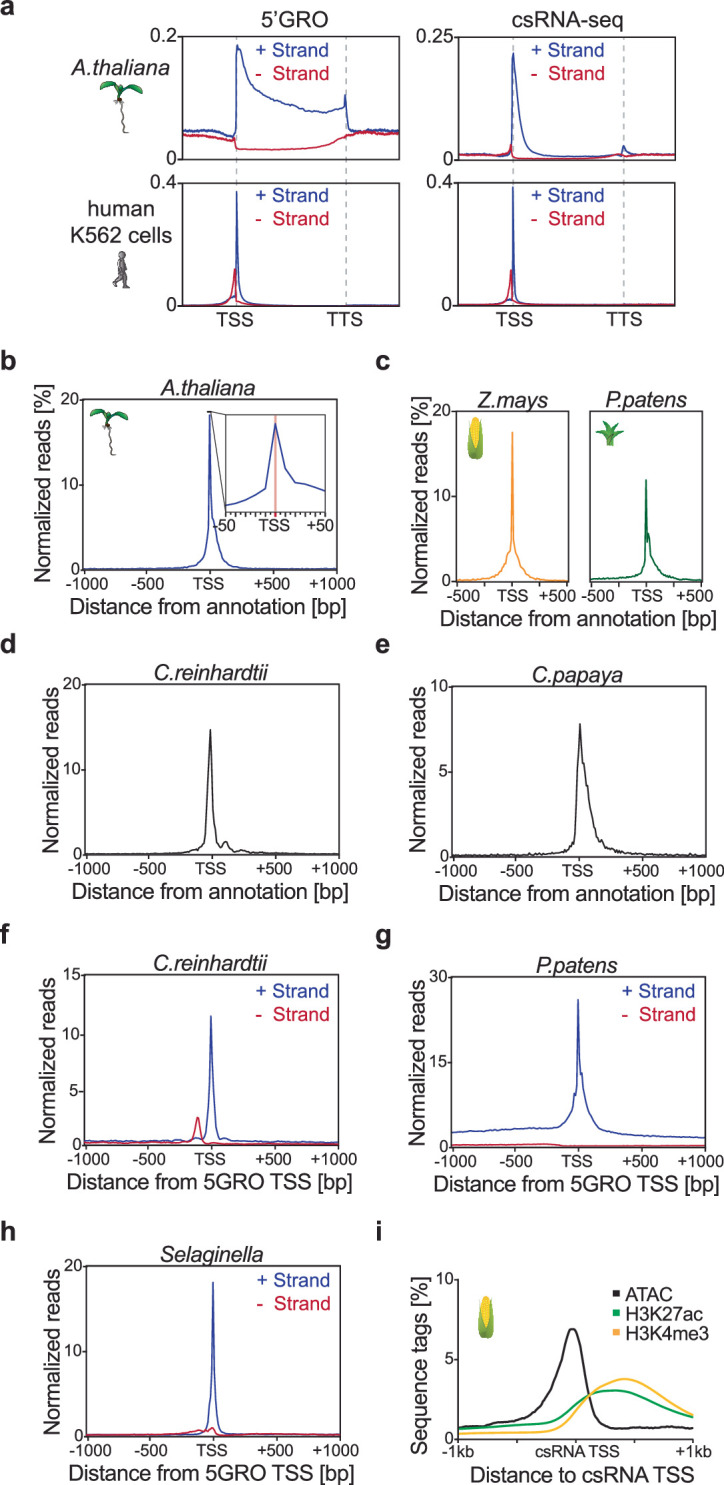
csRNA-seq accurately captures transcription initiation sites (TSSs) across diverse plant species. **a**, Metaplots of 5’GRO-seq or csRNA-seq reads relative to gene annotation start sites (TSS) and ends (Transcription Termination Sites, TTS). **b-e**, Distribution of csRNA-seq TSSs, relative to genome annotations, in *A. thaliana* (**b**), maize and *P. patens* (**c**), *C. reinhardtii* (**d**) and papaya leaves (**e**). **f-h**, Distribution of csRNA-seq TSSs relative to 5’GRO-seq TSSs in *C. reinhardtii* (**f**), *P. patens* (**g**) and *Selaginella* (**h**). **i**, Distribution of open chromatin (ATAC-seq) and histone marks H3K4me3 and H3K27ac from relative to csRNA-seq TSSs in maize leaves.

csRNA-seq accurately captured actively transcribed stable and unstable RNAs (Extended Data Fig. 2) and their TSSs genome wide and at single-nucleotide resolution. As exemplified by the *A. thaliana* ER-type Ca^2+^ ATPase 3 (*ECA3*) locus (Fig. 1c) or unstable primary microRNA (miRNA) 161 (Fig. 1d), csRNA-seq performed similarly to other nascent methods but with less background noise on average (Fig. 1c,d and Extended Data Figs. 3 and 4a). TSSs captured by csRNA-seq were enriched near annotated TSSs genome wide (Fig. 1e and Extended Data Fig. 4b–e). About one-third of the csRNA-seq TSSs mapped in *A. thaliana* leaves were identical to those mapped by 5′ GRO-seq in 6-day-old seedlings, and nearly all were within 200 bp (Fig. 1f)^23^. TSSs identified by csRNA-seq were also similar to those identified by 5′ GRO-seq in *Physcomitrium patens*, *Chlamydomonas reinhardtii* and *Selaginella moellendorffii* (Extended Data Fig. 4f–h).

**Extended Data Fig. 5:**
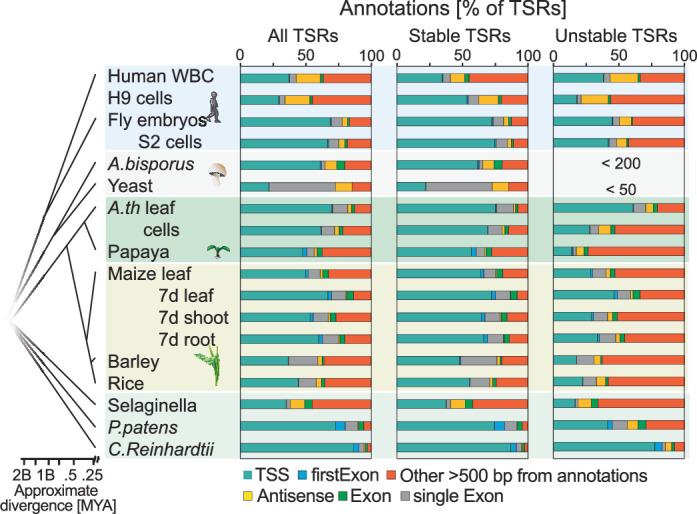
Annotation and features of plant transcription start regions. Annotations of TSRs captured across diverse samples.

To further validate our csRNA-seq TSSs, we examined their association with the chromatin and epigenomic landscape^18,48,49^. As expected for active TSSs, chromatin accessibility (assayed by transposase-accessible chromatin using sequencing (ATAC-seq)) peaked just upstream of csRNA-seq-captured TSSs in both *A. thaliana* (Fig. 1g) and maize (Extended Data Fig. 4i). Histone modifications associated with transcription initiation, such as histone H3 lysine 27 acetylation and H3 lysine 4 trimethylation^50,51^, were found downstream of csRNA-seq TSSs (Fig. 1g and Extended Data Fig. 4i). Regions of transcription initiation were also enriched in genomic regions annotated to be associated with transcription and were mainly found at promoter regions (Fig. 1h). Sites of transcription initiation across plant species revealed a similar pattern to *A. thaliana*, with the majority of TSSs located within annotated promoter regions (Extended Data Fig. 5 and Supplementary Table 2). In addition, csRNA-seq showed efficient and specific enrichment of 5′-capped RNA polymerase II transcripts, with only a small percentage of reads mapping to non-chromosomal regions such as plastids or mitochondria (Fig. 1i). Thus, csRNA-seq accurately captures actively initiated transcripts and their TSSs in diverse plant species and tissues.

**Fig. 2:**
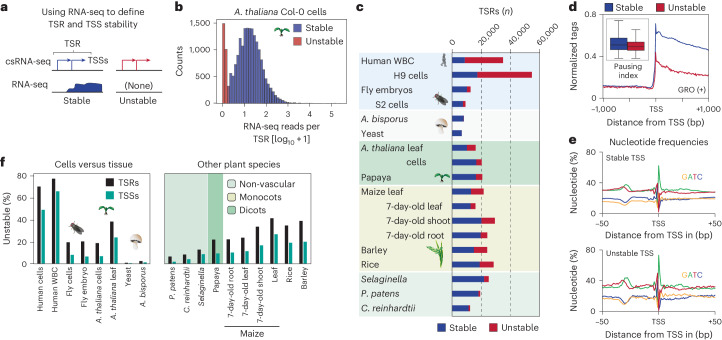
Unstable RNAs are infrequent in plants. **a**, Schema of how transcript stability was determined by integrating total RNA-seq read counts from −100 bp to +500 bp with respect to the major TSSs within TSRs identified by csRNA-seq. **b**, Distribution of RNA-seq reads per million within −100 bp to +500 bp relative to the main TSS of each TSR, plotted as [log_10_ + 1]. **c**, Summary of the number of stable and unstable TSRs in each sample analysed. **d**, GRO-seq signal (positive strand only) in *A. thaliana*^23,30^ in proximity to the TSS of stable and unstable transcripts. Inset: calculated pausing index (reads within −100 bp to +300 bp of the TSS divided by the reads from +301 bp to +3,000 bp; see Methods). Box plots show median values and the interquartile range. Whiskers show minimum and maximum values, excluding outliers. **e**, Metaplot of nucleotide frequency with respect to the +1 TSS as defined by csRNA-seq for stable and unstable transcripts in *A. thaliana*. **f**, Percentage of TSRs and TSSs initiating unstable transcripts across all species and tissues assayed.

**Extended Data Fig. 6:**
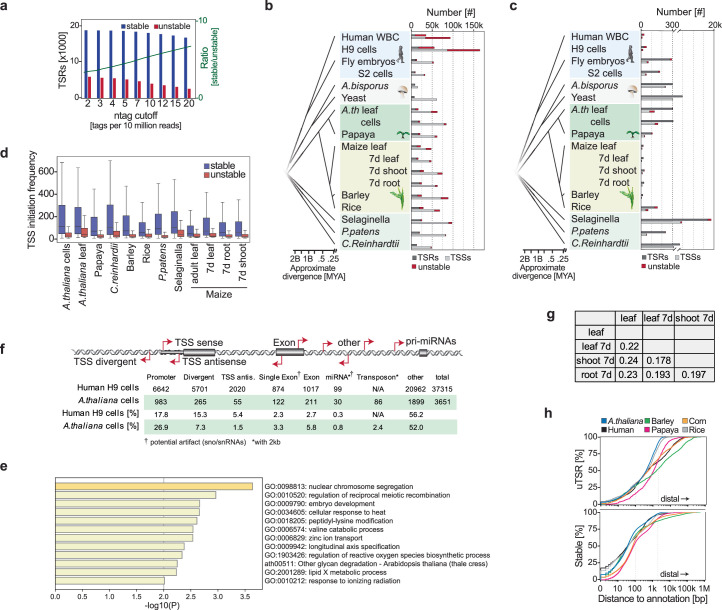
Features of TSRs initiating unstable transcripts. **a**, Titration of TSRs passing the respective ntag threshold (reads per 10 M) as well as separation thereof by initiating transcript stability for *A.thaliana* leaves. **b**, Number of TSSs and TSRs that initiate stable or unstable transcription per species and tissue. **c**, Number of TSSs and TSRs that initiate stable or unstable transcription per species and tissue normalized by total genome size. Note: genome size does not equate to accessible chromatin. **d**, Average RNA polymerase II initiation frequency of TSRs initiating transcripts that are stable or unstable. Boxes show median values and the interquartile range. Whiskers show minimum and maximum values, excluding outliers. **e**, Enrichment analysis on gene sets (gene ontology) of unstable TSRs in *A. thaliana* that annotated to promoters. **f**, Comparison of TSR locations relative to annotations in human H9 cells (gencode.42) and *A. thaliana* Col-0 cells (Araport11). TSS = ± 275 bp of 5’ gene annotation in sense direction; TSS antisense, within the TSS region but antisense; TSS divergent, initiating from −1 to −275bp to the TSS. **g**, Pairwise percent comparison of TSRs that switch between initiating stable and unstable transcripts among maize adult leaves and 7d-old leaves, shoot, and roots. **h**, Number of TSRs initiating stable or unstable transcripts in % relative to genome annotations.

In eukaryotes, most genes display dispersed transcription initiation from multiple TSSs within 20–100 bp in the same promoter or enhancer, classically defined as *cis*-acting DNA sequences that modulate the transcription of genes^52–54^. Therefore, and to avoid implying functionality of studied regulatory regions beyond initiating transcription, we will hereafter jointly refer to all strand-specific individual or clusters of TSSs within 200 bp as transcription start regions (TSRs; Fig. 2a)^54,55^. The number of detected TSSs and TSRs varied from about 60,000 TSSs in 6,500 TSRs in yeast to about 165,000 TSSs in 60,000 TSRs in human H9 cells (Fig. 1b and Supplementary Table 1). Among plant species, we observed a range of TSRs and TSSs, from 12,600 TSRs with 48,000 TSSs in *C. reinhardtii* to 30,000 TSRs with up to 88,000 TSSs in some monocots (for example, barley). Varying analysis parameters only has minor effects on the number of TSRs defined (Extended Data Fig. 6a). Using a high confidence threshold (10 normalized reads or greater), we identified in total >380,000 TSRs with >1.25 million TSSs. This comprehensive atlas provides a valuable resource for studying transcription and gene regulation in plants, spanning over 1.5 billion years of evolution.

### Unstable RNAs are infrequent in plants

csRNA-seq captures active transcription initiation, and thus all RNAs on the continuous scale ranging from highly unstable to very stable (Extended Data Fig. 2). To infer transcript stability, we performed total RNA-seq, which reports stable, steady-state RNAs. We then estimated transcript stability by quantifying total RNA-seq reads near csRNA-seq TSSs (Fig. 2a)^56^. This approach is independent of genome annotations, which vary drastically in quality among the species studied. TSSs of unstable RNAs have few-to-no strand-specific RNA-seq reads downstream (for example, Fig. 1d), whereas stable RNAs are readily detected by RNA-seq (for example, Fig. 1c ref. ^39^). On the basis of the observed bimodal distribution plotting csRNA-seq/total RNA-seq coverage (Fig. 2b) as well as previous analyses^39,43,47^, we defined unstable RNAs as having less than 2 per 10 million RNA-seq reads within −100 bp to +500 bp of the major TSSs within the TSR.

The number of TSRs initiating stable transcripts varied between ~7,000 and 21,000 and was comparatively similar across all species analysed (Fig. 2c and Extended Data Fig. 6b). By contrast, the number and percentage of TSRs and TSSs yielding unstable transcripts varied up to 100-fold. In humans, the majority of TSRs produced unstable transcripts (up to 75%), whereas in fruit flies this frequency was about 20% and in the fungi, yeast and *A. bisporus*, it was less than 2% (Fig. 2c, Extended Data Fig. 6b,c and Supplementary Table 1). In plants, this percentage ranged from 6% to 40%. There was also variability in the proportion of unstable transcripts among different tissues within the same organism, for example, in different maize tissues (Fig. 2c and Extended Data Fig. 6b,c).

Importantly, these numbers probably present the upper limit of unstable transcripts. csRNA-seq is orders of magnitude more sensitive than RNA-seq in detecting recently activated, short or weakly expressed loci^39^. As a result, TSRs that in fact produce stable RNAs could be misclassified as producing unstable RNAs. To mitigate the methodological bias, we focused our analysis, where possible, on simple tissues in near-quiescent states, such as mature leaves and cultured cells. Nevertheless, it is probable that the true number of TSRs producing unstable transcripts is lower than what we are reporting.

**Extended Data Fig. 7:**
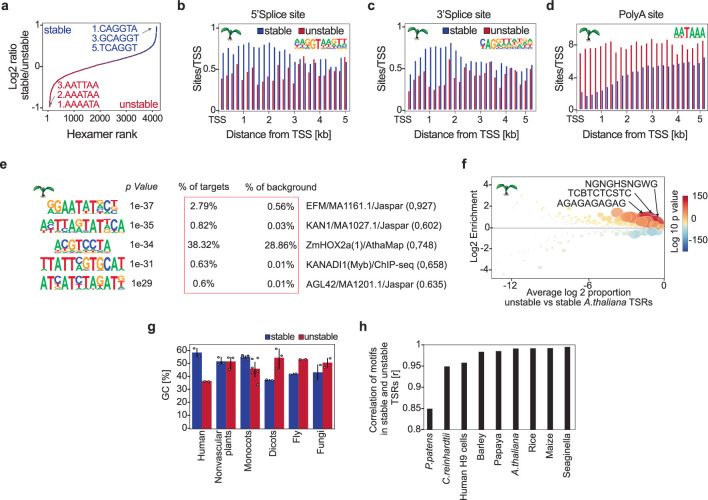
DNA sequence motifs and features of TSRs initiating stable or unstable RNAs. **a**, Rank of all 4096 hexamers by log 2 enrichment relative to transcripts stability within 1 kb downstream of TSSs. **b**, Occurrences of a 5’ splice site downstream of *A. thaliana* TSSs of stable and unstable transcripts. **c**, Occurrences of a 3’ splice site downstream of *A. thaliana* TSSs of stable and unstable transcripts. **d**, Occurrences of a polyadenylation site downstream of *A. thaliana* TSSs of stable and unstable transcripts. **e**, De novo motif analysis using HOMER^28^ of *A. thaliana* cell TSRs regulating unstable transcripts using stable TSRs as background. **f**, Differential motif enrichment analysis of TSRs initiating stable or unstable transcription using CiiiDER^81^. **g**, Average GC content of TSRs in different groups of species. GC content of individual replicates is displayed as dots. Graphs present the mean with SD. **h**, Correlation of DNA sequence motif enrichment scores among TSRs initiating stable and unstable transcription (r-value).

Unstable transcripts could result from premature termination before RNA polymerase II pause release^38^. As csRNA-seq alone cannot discern between this scenario and rapid degradation postinitiation^26^, we integrated published GRO-seq data from *A. thaliana* leaves and seedlings^23,30^. GRO-seq maps engaged RNA polymerases genome wide in a strand-specific manner^34^. Comparing RNA polymerase distribution near TSSs relative to gene bodies (pausing index, reads within −100 bp to +300 bp of the TSS divided by the reads from +301 bp to +3,000 bp^57^) found a modest decrease in RNA polymerase occupancy near TSSs of unstable transcripts compared with stable ones (Fig. 2d). By contrast, TSRs producing unstable RNAs were enriched for TSS-proximal polyadenylation cleavage sites and depleted of RNA splice sites (Extended Data Fig. 7). These findings suggest that, in line with the absence of canonical promoter-proximal pausing in plants^38^, transcript instability is potentially driven by premature degradation related to RNA processing^58,59^ rather than termination dependent on pausing.

Importantly, although unstable transcripts were on average more weakly initiated than stable ones (Extended Data Fig. 6d), the DNA sequence composition surrounding TSRs initiating stable and unstable transcription was highly similar (Fig. 2e). TSRs of both groups had hallmarks of canonical *cis*-regulatory elements, including a TATA box and initiator core promoter signature, emphasizing that these unstable TSRs are not just transcriptional noise. Furthermore, de novo motif analysis^60^ of sequence motifs in proximity to TSSs (−150 bp, +50 bp, relative to the TSS) initiating stable or unstable transcripts also revealed similar occurrences of transcription factor binding sites (*r* > 0.95; Extended Data Fig. 7). These results not only emphasize that both stable and unstable TSSs captured by our method are bona fide TSSs, but also suggest that similar regulatory mechanisms support the initiation of stable and unstable transcripts in plants.

Unstable transcripts are often cell type-specific^14^, which may compromise their detection in complex samples. To address this notion, we compared the detection of TSRs initiating rapidly degraded transcripts across samples with varying cell type complexities. In cultured *A. thaliana* Col-0 cells, approximately 18% of all TSRs initiated unstable transcripts compared with 37% in leaves. About 19% and 20% of TSRs yielded unstable RNAs in fruit fly S2 cells and in 0–12 h embryos, respectively; 0.5% versus 2% were unstable in single-cell yeast versus the multicellular mushroom *A. bisporus*; and 68% and 75% were unstable in human H9 versus white blood cells (Fig. 2f). Thus, there was no substantial difference in the percentage of TSRs or TSSs initiating unstable RNAs in complex versus simpler tissues across kingdoms (Fig. 2f, Extended Data Fig. 6c and Supplementary Table 1). These data argue that the previously reported under-representation of unstable RNAs in plants^23^ is unlikely due to their limited detectability in complex tissues. Although we consistently captured unstable RNAs in diverse plant species, fruit flies and fungi, our data propose that unstable transcription is much less prevalent in all these organisms than in humans.

### Origins of plant unstable transcripts

Studies in vertebrates have described several classes of unstable RNAs, including short, bidirectional eRNAs, promoter-divergent transcripts, and others^41,58,61,62^. As genomic locations of origin were often used to classify these transcript types, rather than functional assays, we compared the genomic locations of unstable RNAs in *A. thaliana* Col-0 cells and human H9 cells for which high-quality reference gene annotations are available. In total, we found 3,651 TSRs initiating unstable transcripts in *A. thaliana* compared with 37,315 in humans. Although this number is about the same when normalizing for genome size, it is important to consider that with 16,527 in *A. thaliana* versus 17,268 in humans, a similar number of stable transcripts was expressed in both species (Fig. 2c).

**Fig. 3:**
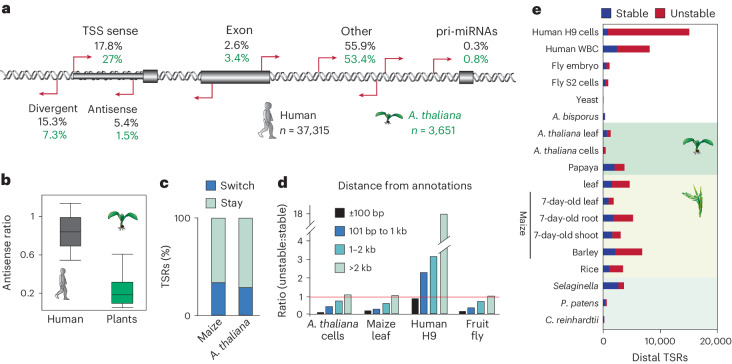
Distinct origins of stable and unstable transcripts in humans, plants and other species. **a**, Classification of TSRs producing unstable transcript genomic sites in human H9 cells and *A. thaliana* Col-0 cells, relative to current annotations (Araport11 or gencode.42). TSS = ±275 bp of 5′ gene annotation in sense direction; TSS antisense, within the TSS region but antisense; TSS divergent, initiating from −1 bp to −275 bp to the TSS. **b**, Ratio of promoter-proximal antisense transcription reveals most plant but not human unstable transcripts to initiate in the sense direction. Ratio of TSRs in antisense to genome-annotated gene 5′ ends (−275 bp to +275 bp relative to the annotated TSS) divided by the number of total TSRs that mapped to annotated TSS. Boxes show median values and the interquartile range. Whiskers show minimum and maximum values, excluding outliers. **c**, Percentage of TSRs that switch between initiating stable and unstable transcripts among *A. thaliana* Col-0 cells and leaves, maize adult leaves, and 7-day-old leaves, shoot and roots. **d**, Number of TSRs initiating unstable divided by stable transcripts relative to distance to genome annotations by regions (1) ±100 bp, (2) 101–1,000 bp, (3) 1,001–2,000 bp and (4) >2,000 bp for *A. thaliana*, fruit fly S2, human cells and maize leaves. **e**, Number of TSRs >2,000 bp from annotations that initiate stable or unstable transcripts. pri-miRNAs, primary miRNAs.

Whereas unstable transcripts from promoter divergent or antisense transcription were prominent in humans, unstable transcripts in plants predominantly originated from promoters in sense (Fig. 3a,b). Approximately 27% of TSRs producing unstable transcripts in *A. thaliana* initiated in the sense orientation from annotated gene 5′ ends, compared with 17.8% in humans (Fig. 3a). These promoters in *A. thaliana* were often tissue-specific but were not enriched for specific pathways or gene sets (Extended Data Fig. 6e). Approximately 7.3% of unstable RNA initiation events were promoter proximal and divergent, compared with 15.3% in human cells (Fig. 3a and Extended Data Fig. 6f). Another 1.5% and 5.4% in *A. thaliana* and humans, respectively, were within 300 bp downstream of the TSS and therefore TSS antisense.

We found that 2.7% of human and 6.6% of *A. thaliana* TSR-producing unstable RNAs annotated to single-exon transcripts such as small nuclear RNA and small nucleolar RNA. These short transcripts are inefficiently captured by total RNA-seq due to their small size and therefore may not be truly unstable^39^. Some TSRs initiating unstable RNAs were found in the proximity of genes encoding miRNAs (Fig. 3a), probably presenting primary miRNA promoters. Only 2.6% of human TSRs and 3.4% *A. thaliana* TSRs producing unstable RNAs were in genic exons.

Therefore, most TSRs that produce unstable RNAs were outside annotated regions in both human H9 cells (55.9%, ~21,000 TSRs) and *A. thaliana* Col-0 cells (53.4%, ~1,950 TSRs) (Fig. 3a). However, as detailed below, many of these ‘distal loci’ in plants—but not humans—also initiated stable transcripts in other tissues. Furthermore, it is important to reiterate that, given the higher sensitivity of csRNA-seq over RNA-seq^39^, many of the promoter sense transcripts classified as unstable could be newly activated genes or non-coding RNAs, suggesting that the true number of unstable RNAs found in plants would be even lower than what we are reporting.

### Many plant TSRs give rise to stable and unstable transcripts

To determine if TSRs can switch between initiating RNAs that are stable or rapidly degraded, we compared transcript stabilities across the different samples of a given species. We found that about 28.4% of TSRs in *A. thaliana* and 33.4% in maize switched in at least one condition, whereas the remainder consistently produced only stable or unstable transcripts (Fig. 3c). Thus, many TSRs can give rise to stable or rapidly degraded transcripts, often in a tissue-specific context, corroborating the notion that RNA stability is largely controlled postinitiation^36,58^.

Given these findings, we also explored the spatial relationship between TSRs and annotations across species. Despite a notable proportion of TSR-initiating unstable transcripts being within 100 bp of annotated gene 5′ ends (28% in *A. thaliana* cells, 50% in maize leaves and 64% in fruit fly S2 cells), proportionally, these regions predominantly generated stable transcripts (Fig. 3d and Extended Data Fig. 6h). Conversely, in humans, a comparable number of TSRs generating stable and unstable transcripts were within 100 bp of annotations.

Across all species examined, the more distal a TSR was from annotated gene 5′ ends, the higher was its likelihood to produce an unstable transcript. However, unlike in humans for which the majority of TSRs within 2 kb of annotations yielded unstable transcripts, most TSRs within this range in plants and flies were stable. Even >2 kb from annotations, close to half the TSRs generated stable transcripts in our plant and fly samples (Fig. 3d). These findings caution against presuming distal transcripts to be inherently unstable; many distal TSRs initiate stable RNAs in plants and thus may be promoters of unannotated genes or non-coding RNAs (Fig. 3d). Indeed, we identified 19,397 distal TSRs in plants that initiated stable RNAs. Together, our results suggest that unannotated promoters and cell-type-dependent stability are probably the major source of apparently unstable transcripts in plants and that bona fide unstable RNAs are much rarer in plants than in humans.

### Canonical vertebrate enhancers are rare in plants

**Fig. 4:**
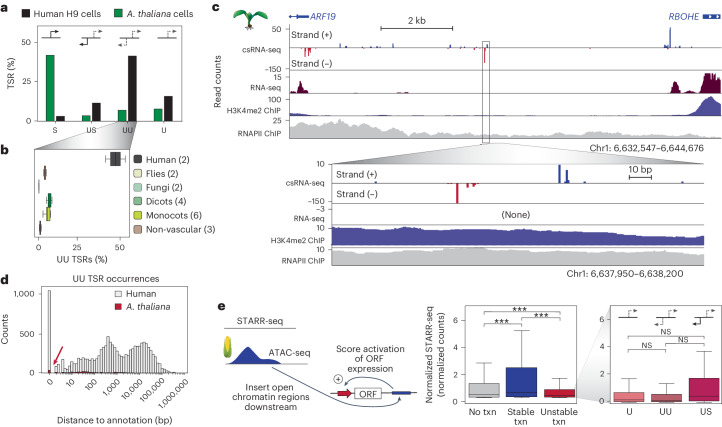
Vertebrate-like enhancers are rare in plants and have less enhancer activity than promoters. **a**, Overview of TSR directionality and type in human H9 cells and *A. thaliana* Col-0 cells. Initiation styles are defined as follows: S, TSR is stable and unidirectional; US, TSR produces an unstable sense transcript and a stable antisense transcript; UU, TSR produces unstable sense and antisense transcripts; and U, TSR is unstable and unidirectional. **b**, Average percentage of bidirectional unstable transcription in samples from humans (H9 cells and WBC), fruit flies (embryos and S2 cells), fungi (*S. cerevisiae* and *A. bisporus*), dicots (*A. thaliana* cells and leaf and papaya), monocots (maize, rice and barley) and non-vascular plants (*Selaginella*, *P. patens*, and *C. reinhardtii*). Boxes show median values and the interquartile range. Whiskers show minimum and maximum values, excluding outliers. Numbers in parentheses indicate number of samples in the group. **c**, Example of 1 of 72 distal TSRs in *A. thaliana* leaves initiating unstable bidirectional transcription. **d**, Distribution of distance to nearest genome annotations for all TSRs initiating unstable bidirectional transcription; annotations in human H9 and *A. thaliana* Col-0 cells. **e**, Overview of the STARR-seq assay (left) that measures the ability of DNA regions, here all open chromatin regions in maize captured by ATAC-seq^18^, cloned downstream of a minimal promoter to enhance its transcription. Enhancer function, as measured by STARR-seq promoter activity (scaled by 100), was subgrouped by csRNA-seq in tissue-defined TSR type (no, stable or unstable transcription initiation). Regions initiating unstable transcription were further subgrouped by their initiation styles (U, UU, US). Boxes show median values and interquartile range, with whiskers showing minimum and maximum values (excluding outliers). One-way ANOVA and Tukey’s honestly significant difference (HSD) test were used. ***P < 0.0005, adjusted *P* value calculated by Tukey’s HSD. Left box plot: no transcription (txn) versus stable (adjusted *P* = 2.665 × 10^−14^), no txn versus unstable (adjusted *P* = 1.356 × 10^−5^) and stable versus unstable (adjusted *P* = 3.486 × 10^−14^). Right box plot: U versus UU (adjusted *P* = 0.1802), U versus US (adjusted *P* = 0.8886), and UU versus US (adjusted *P* = 0.5130). Chr1, chromosome 1; NS, not significant; ORF, open reading frame; RNAPII, RNA polymerase II.

**Extended Data Fig. 8:**
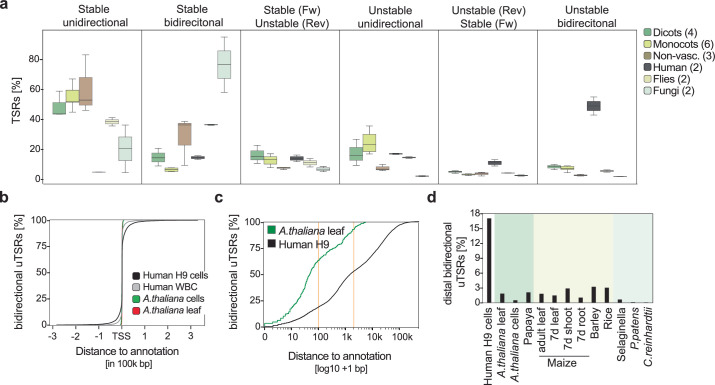
Annotation and abundance of TSRs regulating the initiation of unstable transcripts across species. **a**, TSR types and their relative abundance across diverse species groups. Boxes show median values and the interquartile range. Whiskers show minimum and maximum values, excluding outliers. **b**, Location of bidirectional TSRs initiating unstable transcripts relative to genome annotations in humans (gencode.42) and *A. thaliana* (Araport 11) and **c**, log scale thereof. **d**, Percentage of distal (>2000 bp from annotations) bidirectional TSRs initiating unstable transcription across species and tissues.

Most human promoters and enhancers start transcription in both forward and reverse directions, often from distinct core promoters^36,63^. In contrast to this predominantly bidirectional nature of transcription initiation in humans, we observed that transcription was largely initiated unidirectionally in plants, flies and fungi (Fig. 4a,b and Extended Data Fig. 8a). On average, only 4.7% of TSRs in plants initiated bidirectional unstable transcripts, most of which were promoter proximal (Fig. 4a,b). For instance, in *A. thaliana* leaves, 62% and 91% of bidirectional TSRs were within 100 bp and 2 kb of annotated 5′ ends, respectively (Extended Data Fig. 8b,c).

Although there were definite instances of distal bidirectional initiation of unstable transcripts in plants, reminiscent of canonical mammalian eRNAs (Fig. 4c), they were rare and probably too few to serve as reliable markers for plant enhancers. For instance, only 361 (1.8%) and 72 (0.5%) TSRs in *A. thaliana* Col-0 cells and leaves, respectively, initiated distal bidirectional unstable transcripts. In contrast, 9,318 (17%) of TSRs in human H9 cells initiated bidirectional unstable transcripts that were >2 kb from annotated gene 5′ ends (Fig. 4d and Extended Data Fig. 8d). This difference is not simply due to genome size or gene density: even in monocots with large genomes, the number of distal, unstable and bidirectional initiation events varied between only 400 and 857 events, representing a maximum of 3.2% of TSRs (Extended Data Fig. 8d). As such, distal TSRs initiating bidirectional unstable transcription, a hallmark of vertebrate enhancers^12^, are rare in plants.

### Promoters may function as enhancers in plants

**Extended Data Fig. 9:**
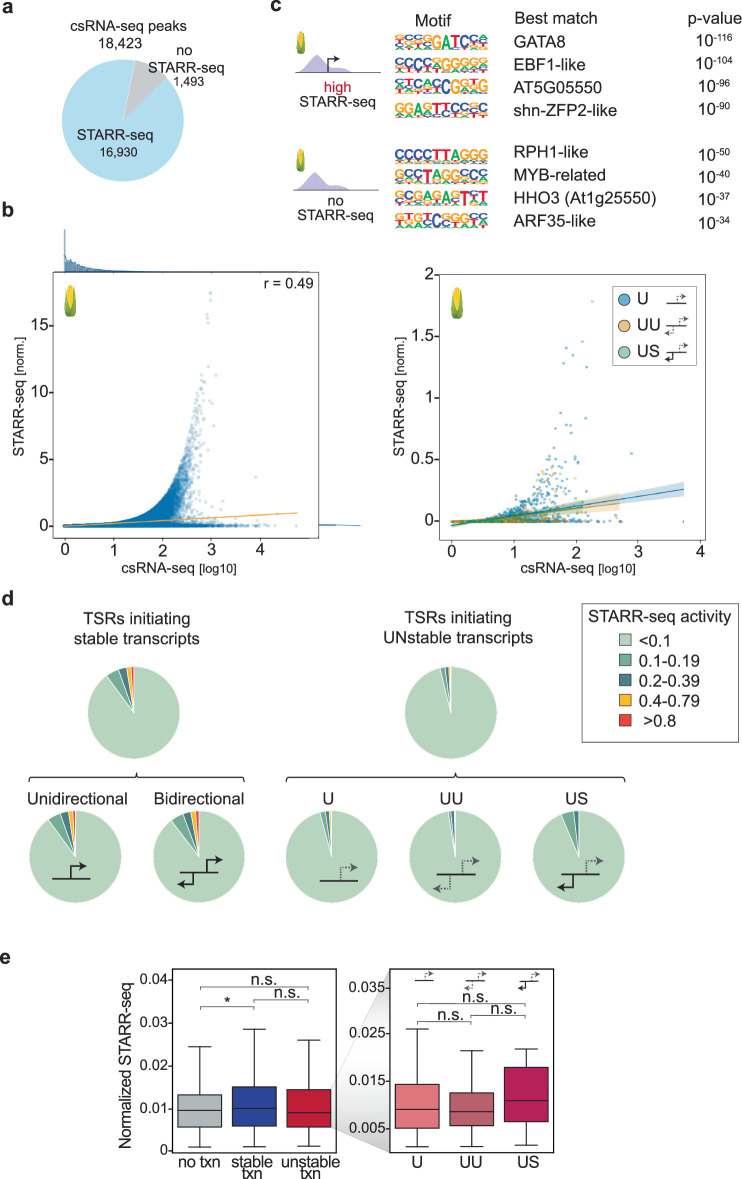
Transcription initiation and STARR-seq enhancer function. **a**, Number or TSRs covered by the STARR-seq input library. **b**, Scatterplot of the STARR-seq activity of all regions in Ricci et al.^8^ maize library with csRNA-seq signal for all loci (left) and TSRs initiating unstable transcription (right). **c**, De novo motifs enriched in regions with high STARR-seq activity vs. none, calculated using HOMER. **d**, STARR-seq enhancer activity of diverse TSR types. **e**, STARR-seq activity of *A. thaliana* genome fragments assayed from Tan et al.^9^ in leaf-derived protoplasts compared to combined *A. thaliana* adult leaf and cell line csRNA-seq TSRs. However, caution needs to be taken with the interpretation of this analysis as the datasets are not tissue-matched and the majority of loci assayed by STARR-seq are in closed chromatin, and thus not assayed by csRNA-seq. Boxes show median values and interquartile range, with whiskers showing minimum and maximum values (excluding outliers). One-way ANOVA and Tukey’s HSD were used; * indicates an adjusted p-value < 0.05 calculated by Tukey’s HSD. Left boxplot: no txn vs stable (adjusted p-value = 0.0442), no txn vs unstable (adjusted p-value = 0.9084), and stable vs unstable (adjusted p-value = 0.4255). Right boxplot: U vs UU (adjusted p-value = 0.6019850), U vs US (adjusted p-value = 0.1535811), and UU vs US (adjusted p-value = 0.0606304).

To explore the functionality of the distal transcription initiation events that we detected in plants, we generated csRNA-seq data matching published STARR-seq data from maize 7-day-old leaves^18^. In this assay, open chromatin regions were cloned downstream of a minimal promoter and their ability to enhance transcription was quantified^64^. The majority (92%) of the csRNA-seq TSRs were covered by the STARR-seq library, indicating effective coverage of the maize genome (Extended Data Fig. 9a). Notably, we found that TSRs initiating stable transcription showed the strongest enhancer activity in plants. Transcription activity, as assayed by csRNA-seq, was overall positively correlated with STARR-seq enhancer activity (*r* = 0.49; Extended Data Fig. 9b). Consistent with these findings, regions with high STARR-seq activity were enriched for binding sites for strong activators like GATA or EBF factors, whereas inactive regions were enriched for binding sites of repressors including *RPH1*, *HHO3* and *ARID* (At1g76110; Extended Data Fig. 9c). These findings suggest that the competence of a regulatory element to recruit RNA polymerase II contributes to its enhancer activity, as assessed by STARR-seq. However, most promoters and even more TSRs producing unstable RNAs showed little STARR-seq enhancer activity (Extended Data Fig. 9d), and STARR-seq enhancer activity was also observed for many open chromatin regions that were transcriptionally inactive (Fig. 4e).

Although vertebrate enhancers are commonly marked by unstable bidirectional transcription (eRNAs), initiation from the upstream STARR-seq promoter in plants was most strongly enhanced by TSRs that initiated stable RNAs (Fig. 4e). TSRs producing unstable RNAs had weak enhancer activity, with TSRs producing vertebrate enhancer-like bidirectional unstable transcripts, on average, showing the weakest activity (UU in Fig. 4e and Extended Data Fig. 9b). Among all TSRs with unstable RNAs, those that had stable transcription initiating from a close TSR upstream showed the highest enhancer activity (US in Fig. 4e). Similar results were obtained using non-tissue-matched *A. thaliana* data^65^ (Extended Data Fig. 9e). Furthermore, in contrast to flies, in which bidirectional but not unidirectional promoters were reported to often act as potent enhancers^37^, both uni- and bidirectional promoters showed similar STARR-seq activity in maize (Extended Data Fig. 9d). Together, these findings underscore the blurred line between the *cis*-regulatory potential of promoters and ‘enhancers’, suggesting that enhancers are a heterogeneous group, and highlight distinct features of plant transcription.

## Discussion

By interrogating initiating transcripts across a wide range of organisms, we discovered that unstable transcripts are rare in plants, and in fact, also in fruit flies and some fungi, compared with mammals. Although the number or percentage of identified unstable transcripts is dependent on analysis thresholds and probably developmental stages, our comparative approach shows that distal bidirectional initiation of unstable transcripts, which is a hallmark of vertebrate enhancers, is rare in plants. Unstable transcripts predominantly originated from unidirectional promoter regions in plants^23^ and we identified numerous distal regulatory elements that initiated stable transcripts, making them bona fide promoters.

These findings suggest that a considerable portion, if not the majority, of unstable RNAs in plants may arise from promoters of either known or unannotated genes or non-coding RNAs^66^, cautioning against presuming transcript stability or enhancers based solely on genome reference annotations. Our comparative analyses also highlight vertebrates as rather distinct in respect to the scale and function of unstable transcription and suggests that the canonical transcribed vertebrate enhancer is just one of many types of enhancer. Moreover, given that diverse types of putative enhancer were observed across all species investigated, this invites speculation that untranscribed enhancers may also play a yet-to-be thoroughly investigated role in vertebrates.

This study should also provide a notable resource to the scientific community. Aside from a comprehensive collection of TSS data paired with total RNA-seq and small RNA-seq (csRNA-seq input) for an array of plant species, tissues and cells, our study shows that csRNA-seq can help to refine genome annotations^67^, readily captures the entire active RNA polymerase II transcriptome in plants and across eukaryotes, and serves as a proof of concept for how csRNA-seq opens up new opportunities to advance our understanding of gene regulation. For instance, csRNA-seq can be readily applied to investigate ongoing transcription in a wide range of scientifically or agriculturally important field samples and tissues, allowing for the decoding of gene regulatory networks implicated in biotic or abiotic stress responses. Caution, however, should be taken in defining transcripts as unstable based on the lack of total RNA-seq signal as the orders-of-magnitude-higher sensitivity of csRNA-seq to detect newly active loci could result in false positives.

Our findings also shed light on the discussion surrounding the role and existence of vertebrate-like eRNAs in plants^24,25,28^ and further blur the line between the concepts of canonical promoters and enhancers. Although distal loci initiating bidirectional unstable transcripts were found in all plant species studied (Fig. 4 and Extended Data Fig. 8d), they were rare and, in some instances, initiated stable transcripts in other tissues or samples from the same plant. Combining csRNA-seq^39^ with STARR-seq^18,64^ showed that genomic regions initiating stable transcription function as stronger enhancers in this assay than those initiating unstable transcription. Intriguingly, among plant TSRs, those resembling mammalian-like enhancers, defined as initiating bidirectional unstable transcription, showed the weakest activating properties in STARR-seq (Fig. 4e and Extended Data Fig. 9). However, we cannot rule out that these regions show enhancer functions by other means not assayed by STARR-seq, such as opening chromatin or impacting spatial or temporal gene activity. In addition, it is important to add that the number of distal TSRs initiating unstable transcription are probably too few to make up all plant enhancers. Although enhancers defined by eRNAs vastly outnumber genes in humans^68^, only a few were observed in plants.

It is notable that many regions that did not initiate transcription in the plant genome, as assayed by csRNA-seq, showed stronger STARR-seq enhancer activity than TSRs producing unstable RNAs (Fig. 4e and Extended Data Fig. 9e). Furthermore, unidirectional plant promoters, on average, displayed similar enhancer activity to bidirectional ones. Contrasting these observations with findings in mammals^13,14^ or flies, in which bidirectional promoters were reported to often act as potent enhancers whereas unidirectional promoters generally cannot^37^, suggests that plant promoters may possess distinct attributes. However, it is also possible that gene regulatory elements form a continuum and that different species or gene regulatory contexts preferentially leverage different parts of it. Although ‘canonical vertebrate enhancers’ with eRNAs may be prevalent in some animals, reports of processed eRNAs^14,16,17^, enhancers functioning as context-dependent promoters^69^ and the important role of enhancers serving as promoters in the birth of new genes^70^ speak to such a continuum and enhancers representing a heterogeneous group of regulatory elements^54,71–73^. If true, this continuum hypothesis would propose that there may also be untranscribed regions or unidirectional promoters that function as enhancers in other species, including humans.

## Methods

### Plant material and growth conditions

*A. thaliana* Col-0 mature leaves were collected from plants grown as described^49^. *A. thaliana* Col-0 suspension cells^74^ were kindly grown by Dr Ashley M. Brooks in 250 ml baffled flasks containing 50 ml of growth medium (3.2 g l^−1^ Gamborg’s B-5 medium, 3 mM MES, 3% [vol./vol.] Suc, 1.1 mg l^−1^ 2,4-dichlorophenoxyacetic acid)^74^ and provided as a frozen pellet. The cultures were maintained at 23 °C under continuous light on a rotary shaker (160 rpm). For *A. thaliana* seedlings, seeds were sterilized using vapour-phase sterilization (exposed to 100 ml bleach + 3 ml concentrated HCl in a vacuum chamber for 3 h) and then approximately 20–40 seeds per plate were sown on 1× MS plates (SKU:092623122; MP Biomedicals) and stratified for 3 days at 4 °C in the dark. Plates were transferred to a growth room and grown for 6 days in long-day conditions (16 h light, 8 h dark). After 6 days, seedlings from each plate were collected into Eppendorf tubes containing a metal ball bearing and immediately flash frozen in liquid nitrogen. Tissue was ground using the Qiagen TissueLyser II, at 30 s^−1^ frequency for 1.5 min twice. RNA was purified using the Zymo Direct-zol RNA MiniPrep kit (R2050). Barley (*Hordeum vulgare*) RNA was isolated by Dr Pete Hedley from embryonic tissue (including mesocotyl and seminal roots; EMB) isolated from grain tissues 4 days past germination^75^. *Physcomitrium* (*Physcomitrella*) *patens* (Gransden) was grown on plates with BCDA medium in a growth cabinet at 21 °C under 16 h light. *S. moellendorffii* was purchased online from Plant Delights Nursery and grown at the window under normal daylight for 1 week before isolating RNA from stems and leaves. *Carica papaya* was purchased from the store and seeds were grown in soil for 6 weeks before leaves were collected. *C. reinhardtii*, which was kindly provided by Dr Will Ansari and Dr Stephen Mayfield (University of California (UC) San Diego), was grown to late logarithmic phase in TAP (Tris–acetate–phosphate) medium at 23 °C under constant illumination of 5,000 lux on a rotary shaker. Adult second and third leaves from *Zea mays* L. cultivar B73 were kindly provided by Dr Lauri Smith (UC San Diego). Plants were grown in 4 inch pots in a greenhouse (temperature, 23 °C–29 °C) without supplemental lighting or humidification (humidity in the 15 h following inoculation ranged between 70% and 90%) year round in La Jolla, CA. RNA from *Z. mays* L. cultivar B73 7-day-old shoot, root and leaves was extracted in the Schmitz Laboratory (University of Georgia) as described in ref. ^18^.

### csRNA-seq library preparation

csRNA-seq was performed as described in ref. ^39^. Small RNAs of ~20–60 nt were size selected from 0.4–3 µg of total RNA by denaturing gel electrophoresis (catalogue number EC68852BOX). The 20–60 nt size limit excludes the smallest steady-state RNA found in these species (62 nt) and 5′-capping selection ensures the capture of RNA polymerase II transcripts, thus enriching initiating RNA polymerase II transcripts^39^. A 10% input sample was taken aside and the remainder was enriched for 5′-capped RNAs. Monophosphorylated RNAs were selectively degraded by 1 h incubation with Terminator 5′-Phosphate-Dependent Exonuclease (TER51020; Lucigen). Subsequently, RNAs were 5′ dephosphorylated through 90 min total incubation with thermostable QuickCIP (M0525L; NEB) in which the samples were briefly heated to 75 °C and quickly chilled on ice at the 60 min mark. Input (small RNA) and csRNA-seq libraries were prepared as described in ref. ^23^ using RppH (M0356; NEB) and the NEBNext Small RNA Library Prep kit (E7560S). RppH cleaves polyphosphates like the 5′ cap, leaving a 5′ monophosphate on RNA that is required for 5′ monophosphate-dependent 5′ adaptor ligation by RNA ligase 1 (see NEBNext kit for details). Libraries were amplified for 11–14 cycles.

### 5′ GRO-seq library preparation

5′ GRO-seq was performed as described by ref. ^23^. Please note that obtained data vary in quality.

### Total RNA-seq library preparation

Strand-specific, paired-end libraries were prepared from total RNA by ribosomal depletion using the Ribo-Zero Gold Plant rRNA Removal Kit (20020599; Illumina). Samples were processed following the manufacturer’s instructions.

### Sequencing information

csRNA-seq libraries were sequenced on an Illumina NextSeq 500 instrument in the Benner Laboratory or, as for the total RNA-seq libraries, using a NovaSeq S6000 at the IGM Genomics Core at UC San Diego. Information on read counts and alignment statistics can be found in Supplementary Table 4.

### Data analysis

A list of genomes and annotations is provided in Supplementary Table 5.

### csRNA-seq data analysis

TSRs, TSSs and their activity levels were determined by csRNA-seq and analysed using HOMER v.4.12 (ref. ^39^). Additional information, including analysis tutorials are available at https://homer.ucsd.edu/homer/ngs/csRNAseq/index.html. TSR files for each experiment were added to the Gene Expression Omnibus data.

csRNA-seq (~20–60 nt) and total small RNA-seq (input) sequencing reads were trimmed of their adaptor sequences using HOMER (‘batchParallel.pl ‘homerTools trim -3 AGATCGGAAGAGCACACGTCT -mis 2 -minMatchLength 4 -min 20’ none -f {csRNA_fastq_path}/*fastq.gz’) and aligned to the appropriate genome using Hisat2 (ref. ^76^) (‘hisat2 -p 30 --rna-strandness RF --dta -x {hisat2_genome_index} -U {path_rimmed_csRNA or sRNA} -S {output_sam} 2> {mapping_stats}’). Hisat2 indices were generated for each genome using ‘hisat2-build -p 40 genome.dna.toplevel.fa {Hisat2_indexfolder}’ except barley, which required addition of ‘--large-index’. HOMER genomes were generated using ‘loadGenome.pl -name {Homer_genome_name} -fasta {species.dna.toplevel.fa} -gtf {species.gtf}’. Only reads with a single, unique alignment (mapping quality ≥ 10) were considered in the downstream analysis. The same analysis strategy was also used to reanalyse previously published TSS profiling data to ensure the data were processed in a uniform and consistent manner, with the exception of the adaptor sequences, which were trimmed according to each published protocol. Tag directories were generated as described in the csRNA-seq tutorial. We automated the process for all species by first generating an infofile.txt and then generating them in a batch as follows.

for species in species_list:

 !ls $sam_path/*.sam> $sam_path'samNames.txt'#list all sam files and save them to the list

 samNames = pd.read_csv(sam_path +'samNames.txt', sep='\t', names = ['samFile']) #read in file and name the column of interest

 tagDirName = samNames['samFile'].str.split('(-r[1|2|3|4|5|6|7|8|9|10|11])', n=1, expand = True) #generate a new column with the truncated name = the name I want for the tagdir

 tagDirName.columns = ['1', '2','toss'] #name columns

 tagDirName_concat = tagDirName[['1','2']].apply(lambda x: None if x.isnull().all() else ';'.join(x.dropna()), axis=1) #no avoid empty rows give nan

 tagDirName_concat = pd.DataFrame(tagDirName_concat, columns = ['tagDirs']) #remake df

 tagDirName_concat['tagDirs'] = tagDirName_concat['tagDirs'].str.replace('.sam','').str.replace(sam_path,'').str.replace('/',tagdir_path).str.replace(';','') #first remove sam from files that lack-r, then remove the fastq path but add the tagDirs path

 mkDirsFile = pd.concat([tagDirName_concat['tagDirs'], samNames['samFile']], axis=1, Sort=False) #save as a txt for the next command but ignore the header and index

 mkDirsFile.to_csv(infoFile_path, sep = '\t', index = False, header=False)

 mkTagDirs = f'batchMakeTagDirectory.pl {infoFile_path} -cpu 50 -genome {genome} -omitSN -checkGC -fragLength 150 -single -r'

 !{mkTagDirs}

**Extended Data Fig. 10:**
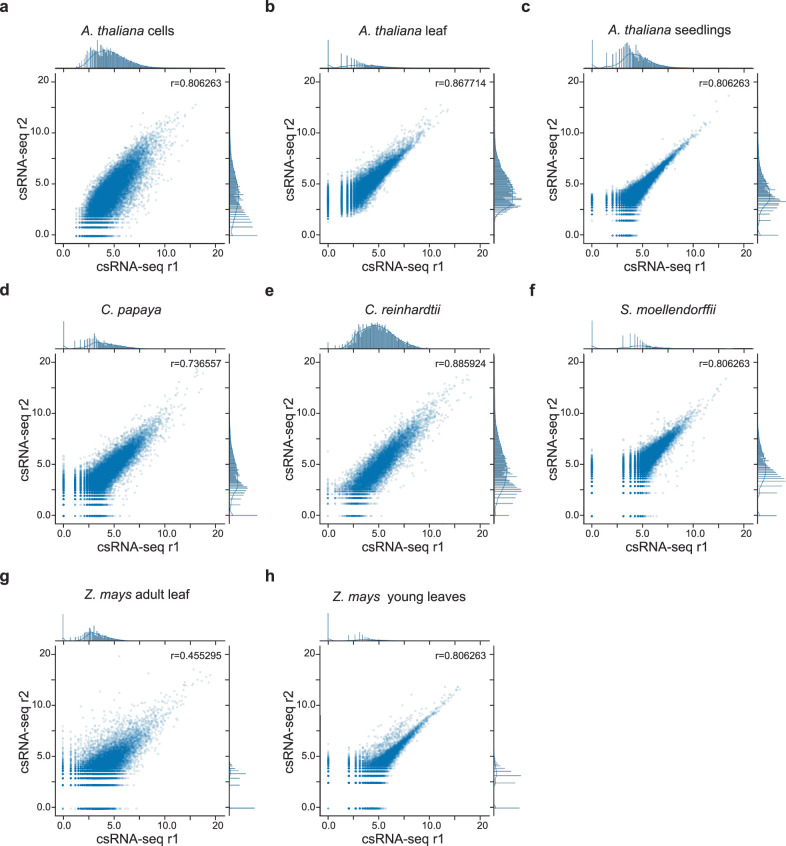
Variance of biological csRNA-seq replicates. Scatterplots comparing rlog tag normalization similarity between biological replicates for **a**, *A. thaliana* cells, **b**, *A, thaliana leaf*, **c**, *A. thaliana* seedlings (6 days), **d**, *C. papaya*, **e**, *C. reinhardtii*, **f**, *S. moellendorffii*, **g**, *Z. mays* adult leaf and, **h**, *Z. mays* young leaves (7 days).

The number of biological replicates generated for each species and sample type are as follows: *A. thaliana* cells, *n* = 2; *A. thaliana* leaves, *n* = 2; *A. thaliana* 6-day-old seedlings, *n* = 2; *C. papaya*, *n* = 2; *C. reinhardtii*, *n* = 2; fruit fly embryos, *n* = 1; fruit fly S2 cells, *n* = 1; *H. vulgare*, *n* = 1; *P. patens*, *n* = 1; *S. moellendorffii* stem and leaves, *n* = 2; *Z. mays* adult leaf, *n* = 2; *Z. mays* young leaves, *n* = 2; *Z. mays* shoot, *n* = 1; and *Z. mays* root, *n* = 1. Comparisons among the biological replicates are shown in Extended Data Fig. 10.

TSSs and TSRs were analysed in this study. TSRs, which comprise one or several closely spaced individual TSSs on the same strand from the same regulatory element (that is, ‘peaks’ in csRNA-seq), were called using findcsRNATSS.pl^39^ (‘findcsRNATSS.pl {csRNA_tagdir} -o {output_dir} -i {sRNA_tagdir} -rna {totalRNA_tagdir} -gtf {gtf} -genome {genome} -ntagThreshold 10’). findcsRNATSS.pl uses short input RNA-seq, total RNA-seq (Ribo0) and annotated gene locations to find regions of highly active TSSs and then eliminate loci with csRNA-seq signals arising from non-initiating, high-abundance RNAs that nonetheless are captured and sequenced by the method (for more details, see ref. ^39^). Replicate experiments were first pooled to form meta-experiments for each condition before identifying TSRs. Annotation information, including gene assignments, promoter distal, stable transcript and bidirectional annotations are provided by findcsRNATSS.pl. To identify differentially regulated TSRs, TSRs identified in each condition were first pooled (union) to identify a combined set of TSRs represented in the dataset using HOMER’s mergePeaks tool using the option -strand. The resulting combined TSRs were then quantified across all individual replicate samples by counting the 5′ ends of reads aligned at each TSR on the correct strand. The raw read count table was then analysed using DESeq2 to calculate normalized rlog-transformed activity levels and identify differentially regulated TSRs^77^.

TSSs were called using getTSSfromReads.pl (‘getTSSfromReads.pl -d {csRNA_tagdir} -dinput {sRNA_tagdir} -min 7 > {output_file}’^39^). To ensure high-quality TSSs, at least 7 per 10^7^ aligned reads were required and TSSs were required to be within called TSRs (subsequently filtered using mergePeaks ‘mergePeaks {TSS.txt} {stableTSRs.txt} -strand -cobound 1 -prefix {stable_tss}’ or ‘mergePeaks {TSS.txt} {unstableTSRs.txt} -strand -cobound 1 -prefix {unstable_tss}’). Furthermore, TSSs that had higher normalized read density in the small RNA input sequencing than csRNA-seq were discarded as a likely false positive TSS location. These sites often include miRNAs and other high-abundance RNA species that are not entirely depleted in the csRNA-seq cap-enrichment protocol. In most cases, TSRs were analysed (that is, to determine motifs or describe the overall transcription activity of regulatory elements) but, when indicated, single-nucleotide TSS positions were independently analysed (that is, to determine motif spacing to the TSS).

Annotation of TSS or TSR locations to the nearest gene was performed using HOMER’s annotatePeaks.pl program using GENCODE as the reference annotation^60^.

Genomic positions with sequence tags were extracted from HOMER tagDirectories using getTSSfromReads.pl with parameter -min 0 using published data^36,63^ and data generated in this study. These positions were then merged with TSRs (mergePeaks -strand) and number of regions and tags were counted (Extended Data Fig. 3a,b). Histograms were generated using seaborn histplot with log_10_, binwidth = 0.1 (Extended Data Fig. 3c–f).

TATA box motif distribution plots (Extended Data Fig. 3g,h) for tags within or outside of called TSRs were generated using HOMER (annotatePeaks.pl {file} tair10 -size 150 -hist 1 -m ~/HOMER/motifs/CPE/TATAWAAR.motif). Distance was calculated for each unique nucleotide position (0).

Strand-specific and other IGV and genome browser files were generated using ‘makeUCSCfile {tag_directory_name} -strand + -fragLength 1 -o {tag_directory_name}.bedGraph’ where the tag_directory could be csRNA-seq or 5′ GRO-seq data from any species or tissue.

### 5′ GRO-seq and GRO-seq analysis

Published and generated 5′ GRO-seq and GRO-seq data were analysed as described for csRNA-seq and small RNA-seq above. 5′ GRO-seq peaks were called using HOMER’s ‘findPeaks {5GRO_tagdirectory} -i {GRO_tagdirectory} -style tss -F 3 -P 1 -L 2 -LP 1 -size 150 -minDist 200 -ntagThreshold 10 > 5GRO_TSRs.txt’. A detailed explanation of each parameter can be found at http://homer.ucsd.edu/homer/ngs/tss/index.html.

### RNA-seq analysis

Paired-end total ribosomal, RNA-depleted RNA-seq libraries were trimmed using skewer (‘time -p skewer -m mp {read1} {read2} -t 40 -o {trimmed_fastq_output}’)^78^ and aligned using Hisat2 (ref. ^76^) to ensure all data were processed as similarly as possible (‘hisat2 -p 30 --rna-strandness RF --dta -x {hisat2_index} -1 {trimmed_RNAseq_R1} -2 {trimmed_RNAseq_R2} -S {output_sam} 2> {mapping_file}’). In this article, total RNA-seq was exclusively used to determine RNA stability as described in the csRNA-seq analysis.

### Chromatin immunoprecipitation with massively parallel DNA sequencing analysis

Tag directories were generated for paired-end sequenced chromatin immunoprecipitation (ChIP-seq) libraries as described for total RNA-seq. Peaks were called using HOMER’s **‘**findPeaks {ChIP_tagdir} -i {ChIP_inout_tagdir} -region -size 150 -minDist 370 > ChIP_peaks.txt’.

Quantification of histone modifications associated with each TSS was performed from +1 bp to +600 bp to capture the signal located just downstream from the TSS. When reporting log_2_ ratios between read counts, a pseudocount of ‘1 read’ was added to both the numerator and denominator to avoid dividing by 0 errors and buffer low intensity signal.

### ATAC-seq analysis

ATAC-seq data were analysed as described for csRNA-seq but trimmed using CTGTCTCTTATACACATCT.

### Motif correlation of stable and unstable TSRs

Motifs were defined using HOMER and our 151-motif library using stable or unstable TSRs as foreground and the other as background (‘findMotifsGenome.pl {stable_TSS_file} {species_fa} {species_tss}_stable/ -bg {UNstable_TSS_file} -mask -p 40 -size -150,50 -mset all -S 15 -len 10 find MotifsGenome.pl {UNstable_TSS_file} {species_fa} {species_tss}_UNstable/ -bg {stable_TSS_file} -mask -p 40 -size -150,50 -mset all -S 15 -len 10’). Frames were concatenated and the correlation calculated using the pandas.corr function (https://zenodo.org/record/7794821#.ZD1rA3bMKUk).

### Transcript stability switch analysis

Transcript stability was determined as unstable if <2 reads per 10^7^ total RNA-seq reads were within −100 bp, +500 bp of the main TSS of the TSR. In *A. thaliana* we compared cells and adult leaves to identify transcripts that had differential stability among the conditions; in maize we used adult leaves, 7-day-old seedling leaves, 7-day-old seedling roots and 7-day-old seedling shoots. For the plots (Fig. 3c; sns.pointplot) we limited our analysis in maize to 7-day-old shoot versus root.

### Mapping statistics calculation

All outputs (‘<2’) from Hisat2 were copied into a mappingstats folder and summarized using the following custom code:

mappingStats_dict = {"Library":[],"Reads":[], "Adapter reads":[],"Aligned 0 times":[],"Aligned 1 time":[],"Aligned >1 times":[], "Adapters %":[],"Aligned 0 times %":[],"Aligned 1 time %":[],"Aligned >1 times%":[],"Alignment rate":[]}

for mapping_file in os.listdir('mappingstats_folder'):

- if mapping_file.endswith('_mappingstats.txt'):
  - mapping_frame = pd.read_csv(mappingstats_folder + mapping_file, sep='\t')
  - library = mapping_file.split('.fastq')[0]
  - reads = (mapping_frame.loc[0][0]).split(' ')[0]
  - aligned_0 = (mapping_frame.loc[2][0]).split(' (')[0].split(' ')[-1]
  - aligned_0percent = (mapping_frame.loc[2][0]).split('(')[1].split(')')[0]
  - aligned_1 = (mapping_frame.loc[3][0]).split(' (')[0].split(' ')[-1]
  - aligned_1percent = (mapping_frame.loc[3][0]).split('(')[1].split(')')[0]
  - aligned_more = (mapping_frame.loc[4][0]).split(' (')[0].split(' ')[-1]
  - aligned_morePercent = (mapping_frame.loc[4][0]).split('(')[1].split(')')[0]
  - rate = (mapping_frame.loc[5][0]).split(' ')[0]
  - ### also read out adapter dimers ###
  - species = mapping_file.split('_')[0] + '_' + mapping_file.split('_')[1].split('-')[0]
  - trimmed_lengths_file = '/data/lab/duttke/labprojects/plants_2023/data/' + species + '/fastq/csRNA/' + mapping_file.split('_mappingstats.txt')[0] +'.fastq.gz.lengths'
  - trimmed_lengths_frame = pd.read_csv(trimmed_lengths_file, sep='\t')
  - adapter_dimers_reads = trimmed_lengths_frame.loc[0][1]
  - adapters_percent = round(float((trimmed_lengths_frame.loc[0][2]).split('%')[0]),2)
  - mappingStats_dict["Library"].append(library)
  - mappingStats_dict["Reads"].append(reads)
  - mappingStats_dict["Adapter reads"].append(adapter_dimers_reads)
  - mappingStats_dict["Aligned 0 times"].append(aligned_0)
  - mappingStats_dict["Aligned 1 time"].append(aligned_1)
  - mappingStats_dict["Aligned >1 times"].append(aligned_more)
  - mappingStats_dict["Adapters %"].append(adapters_percent)
  - mappingStats_dict["Aligned 0 times %"].append(aligned_0percent)
  - mappingStats_dict["Aligned 1 time %"].append(aligned_1percent)
  - mappingStats_dict["Aligned >1 times %"].append(aligned_morePercent)
  - mappingStats_dict["Alignment rate"].append(rate)
- mappingStats_dict_frame = pd.DataFrame(mappingStats_dict)
- mappingStats_dict_frame = mappingStats_dict_frame.sort_values(by=['Library'])
- mappingStats_dict_frame.to_csv('summary_mappingStats.tsv', sep = '\t')

### Histograms and annotation of TSS to captured reads

Histograms showing csRNA-seq or other data relative to known TSS were generated using ‘annotatePeaks.pl {known TSS} {species_homer_genome (for example TAIR10)} -strand + -fragLength 1 -size 100 -d {species_tagdirectory (for example P.patens_csRNAseq)} -raw > output.tsv’. Known TSSs were extracted from .gtf files using ‘parseGTF.pl {species_gtf_file} tss > {species}_genes.tss’.

Histograms showing called TSS by csRNA-seq or 5′ GRO-seq relative to one another or ‘known TSS’ were generated using ‘annotatePeaks.pl {reference or ‘Known TSS’} {species_homer_genome (for example TAIR10)} -p {2nd TSS file, that is csRNA-seq TSS} -size 2000 -hist 1 -strand +> output.tsv’.

### Tag distribution histograms

Genome-wide read counts were obtained using HOMER2’s^79^ getTSSfromReads.pl script (getTSSfromReads.pl -d {tagdir} -min 0 > {output_tags.txt}). These read counts were then overlaid on called peaks in a strand-specific manner using the mergepeaks command (mergepeaks {output_tags.txt} {called_peaks.txt} -strand > {merged_output_tags.txt}), and the distributions were counted and plotted using seaborn’s histplot function.

### Hexamer analysis

All possible combinations of 6 nt sequences (hexamers) were generated as follows:

 nucleotides = ["A", "G", "C", "T"]

 hexamers = []

 for i in product(nucleotides, repeat = 6):

 hexamers.append(''.join(i))

We then extended TSR peaks from +1 kb to +3 kb for each species and split them based on the stability of initiating transcripts. Occurrences of each hexamer were counted in the stable versus unstable sequences and normalized by the respective number of TSRs. A stability ratio was calculated by dividing the normalized stable hexamer account by the normalized unstable hexamer account. We then ranked the hexamers based on their enrichment in TSRs initiating unstable transcripts over stable ones: 1 being unstable and 4,096 being stable.

### RNA processing-related motif finding

Single-nucleotide TSSs were extended to 5 kb using the adjustPeakFile.pl script (adjustPeakFile.pl {stable/unstableTSS.txt} -size 0,5000 > {outputFile1}). Subsequently, DNA sequences were extracted using HOMER’s extract command (homerTools extract {outputFile1} {genome.fa} > {outputFile2}) and converted into a fasta format. Putative motifs, including the poly(A), 5′-splice and 3′-splice sites, were annotated using the findMotifs.pl script (findMotifs.pl {outputFile2.fa} fasta {output_directory}/ -len 10 -mask -norevopp -find {motifs_of_interest} > {output_motifs}). The instances of motifs were summed up and divided by the number of input TSSs to normalize counts to motif occurrences per TSS and the data were plotted using seaborn.

### Pausing index

The pausing index was calculated as described^57^ using reads near TSRs (−100 bp to +300 bp) divided by those found downstream in the region of +301 bp to +3 kb, relative to the major TSS of the TSR.

### Gene Ontology analysis

Gene Ontology analysis was performed using METASCAPE^80^ for transcripts annotated within 500 bp downstream of the main TSS of TSRs.

### STARR-seq analysis

csRNA-seq data were generated from analogous tissue as used for STARR-seq (GSE120304_STARR_B73_enhancer_activity_ratio.txt.gz) by ref. ^18^ as described above. For compatibility reasons, this analysis thus used the *Z. mays* AGPv4 reference genome and *Z. mays* AGPv4.38 genome annotation instead of maize 5.5. STARR-seq library fragments of 1–50 bp were removed from the analysis as these short fragments disproportionally showed no enhancer activity, whereas longer fragments of the same locus did. csRNA-seq TSRs were defined as described above and merged with the STARR-seq peaks (mergePeaks) to identify overlaps. As sometimes several STARR-seq peaks fell within one TSR, we next corrected the STARR-seq values by linking each mergedPeak identifier with the sum of STARR-seq peaks that fell within the peak. Next, we normalized this value by the length of the peak to obtain a STARR-seq value per base pair for each merged peak and added the csRNA-seq values and TSR stability. To calculate the *P* values for the box plots, we used the pairwise_tukeyhsd function from the statsmodels python package.

### Reporting summary

Further information on research design is available in the Nature Portfolio Reporting Summary linked to this article.

## Supplementary information

Reporting Summary

Supplementary Tables 1–5 Supplementary Table 1–Overview of TSR statistics. Supplementary Table 2–Overview of TSR and TSS annotation stats. Supplementary Table 3–Overview of all data generated and analyzed. Supplementary Table 4–Sequencing and mapping stats of all generated data. Supplementary Table 5–Genomes and annotations.

## Data Availability

All raw and processed data generated for this study can be accessed at NCBI Gene Expression Omnibus accession number GSE233927 and browsed at https://labs.wsu.edu/duttke/mcdonaldbr_ernaplants_2024/. All data generated and analysed are summarized in Supplementary Table 3.

## Code availability

Code used to analyse data in this article has been described in the Methods or is available from the following repositories: HOMER (http://homer.ucsd.edu/) and MEIRLOP (https://github.com/npdeloss/meirlop).
